# A universe of uncertainty hiding in plain sight

**DOI:** 10.1073/pnas.2218530120

**Published:** 2023-01-03

**Authors:** Per Engzell

**Affiliations:** ^a^UCL Social Research Institute, University College London, London WC1H 0AA, United Kingdom; ^b^Nuffield College, University of Oxford, Oxford OX1 1NF, United Kingdom; ^c^Swedish Institute for Social Research, Stockholm University, Stockholm SE-106 91, Sweden

In an impressive effort, Breznau et al. (1) (henceforth, BRW) report a many-analyst collaboration where multiple teams were involved in analyzing the same data and hypothesis: that immigration undermines public support for social policy. Like other such studies (2, 3), the results show considerable variation and are sure to ignite debate. The message is clear: Social scientists must be principled about analytical choices, transparent about their data and procedures, and humble about uncertainty. Analyses such as this have much to teach us about advancing those aims.

Many readers will find BRW’s “hidden universe of uncertainty” harrowing and ask whether we should trust social science at all. Such an implication would be overwrought for several reasons.

The study tests not one but several hypotheses. BRW collected 2 measures of immigration (4 including alternative sources) and 6 measures of policy support. The outcome variables span various domains: jobs, healthcare, pensions, unemployment, redistribution, and housing. Tested models include various combinations of within- and between-country variation, different countries and years, and so on. Given the variety of implied hypotheses, it would be remarkable if results did not vary.

Despite this variation, the study successfully replicates a published null finding. BRW chose their research question because it is “influential, long standing, and typical” (1). But according to the earlier study that BRW replicate (4), results “mostly fail to support” the hypothesis. BRW do not correct for multiple hypothesis testing, but most of their results span a narrow range around zero. As a replication, it is not clear that this should count as a failure and it may well count as a success.

**Fig. 1.**
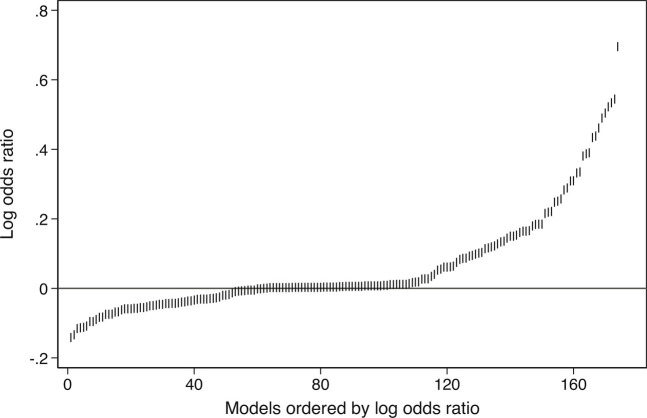
Variation in reported results from Brady and Finnigan (4) which Breznau et al. (1) replicate. Point estimates for 3 variables: percent foreign born, net migration, and change in percent foreign born. Total number of estimates n = 174. Just like in Breznau et al. (1), results span coefficients that are negative, positive, and indistinguishable from zero. Positive log odds ratios imply an association opposite of the hypothesized negative effect of immigration on support for social policy. Odds ratios as reported by Brady and Finnigan are not directly comparable with average marginal effects as reported by Breznau et al.

Much variation is within results from a given team. BRW’s headline finding conflates within- and between-team variation. That results vary across specifications is routine and routinely reported. In fact, results in the original study (4) span a similarly broad range (Fig. 1). It is therefore contestable that variation “remains hidden when considering a single study in isolation” (1).

The data are inadequate for the hypothesis. Given the causal nature of the hypothesis and observational nature of the data, there are inevitable limitations to the conclusions that can be drawn. Moreover, the inherent difficulty of measuring human attitudes is often underappreciated. One team “conducted preliminary measurement scaling tests, concluded that the hypothesis could not be reliably tested, and thus, did not design or carry out any further tests” (1). With more suitable data, one wonders whether the results had not shown greater convergence or at least more meaningful variation.

Limitations notwithstanding, BRW’s study is a landmark in crowdsourced open science. As the authors note, the underspecified nature of hypotheses and identification is representative of much published work. The questions raised in this comment do not diminish the challenges social scientists face in making their discipline more credible. Researcher degrees of freedom remain an important and underrecognized source of uncertainty. Recognizing it should not, however, detract from underlying challenges that may turn out to matter as much if not more: theory, measurement, and causal inference (5–10).
